# Failure Analyses on a Flexible Anode Cathodic Protection System in a Station

**DOI:** 10.3390/ma17020291

**Published:** 2024-01-06

**Authors:** Wenhui Liu, Runyao Chang, Xian Li, Yanxia Du, Jianhua Liu

**Affiliations:** 1 School of Materials Science and Engineering, Beihang University, Beijing 100191, China; sxlwh128@163.com; 2 PipeChina Institute of Science and Technology, Tianjin 300450, China; 3 Institute for Advanced Materials and Technology, University of Science and Technology Beijing, Beijing 100083, China

**Keywords:** cathodic protection, flexible anode, failure analysis, cable breaking, electronic shorting

## Abstract

Flexible anodes are a common form of anode ground bed for the cathodic protection of buried pipes in station areas, especially in new stations. In most cases, flexible anode ground beds could obtain uniform potential distribution and good protection. However, in the process of operation, there are also failure conditions such as anode body or cable breakage, electronic shorting between anode and pipes and other situations, resulting in poor protection. How to troubleshoot failures has become a difficult problem restricting the application of flexible anodes in actual production. In this paper, the failures of a flexible anode cathodic protection system in a station were assessed and analyzed in detail. The main reasons for the failures were the electronic shorting between the flexible anode and buried pipe in local area and the breaking of a partial flexible anode. The troubleshooting methods for two kinds of failures were explored. By measuring the potentials of both the flexible anode and pipes in different areas and the excavation test, the location of electronic shoring was determined. And through measuring the grounding resistance of the flexible anode and excavation test, the breaking location of the flexible anode was found. By repairing the failure sites, the failed system was restored to normal, which could provide guidance for the failure analyses of the flexible anode cathodic protection system.

## 1. Introduction

Cathodic protection mechanisms are used to reduce the potential difference between the local anodes and cathodes to zero by cathodic polarization, resulting in a zero corrosion current flow [1,2,3]. Cathodic protection can be applied to a large network of buried structures and grounding systems. Using impressed current cathodic protection, corrosion is mitigated through a well-designed approach that determines the size, quantity, and location of anodes. In China, this method is commonly referred to as regional cathodic protection. Regional cathodic protection has been applied to buried steel pipes and other metal structures in oil and gas stations to protect them from corrosion, which means protecting all the buried metal structures with electronic connections as a whole by optimizing the distribution of anode ground beds and accurately evaluating the current requirements [4,5,6]. This engineering practice has proved that the combined application of regional cathodic protection and anticorrosion coating can effectively avoid corrosion hazards [7]. In regional cathodic protection, the design and selection of the anode ground bed are the key to achieving ideal protection effects [8,9]. At present, there are generally three kinds of ground beds, including deep well anode, shallow buried distributed anode, and flexible anode [10]. The flexible anode cathodic protection system is an impressed current cathodic protection. The flexible anode is connected to the positive terminal of the power supply from which the current flows out along the cables to flexible anode and then flows from the flexible anode through the soil into the pipes to provide cathodic protection. Extending flexible anodes parallel to the pipeline allows for the uniform distribution of protection potential and the good protection effect of the flexible anode cathodic protection system, and therefore it has been widely used in the regional cathodic protection of stations, especially for new stations [11,12,13]. There are two types of flexible anodes. The first-generation flexible anode was a conductive polymer type flexible anode from the Raychem Company (Menlo Park, CA, USA). Later, the second generation of flexible anodes with MMO/Ti wire as the anode core material was developed [14]. The flexible anode is a flexible, cable-shaped auxiliary anode with a diameter of only about 3~5 cm. The small diameter and related mechanical resistance of the flexible anode, usually installed with a significant length in the vicinity of the installation, increase the risk of failure. In actual use, the breakage of the flexible anode body or cables and other failures occurred sometimes. Lu et al. [15] pointed out in the literature that Ti wire in the flexible anode was easy to break in the field laying and service process, due to non-standardized operation or rough laying construction. However, this disconnection is difficult to observe from the external surface of the anode. Liu et al. [16] reported the case of flexible anode disconnection in the station field. The breakpoint area of the flexible anode cable was found by using Radiodetection (Bristol, UK) RD8000 measuring equipment. RD8000 utilizes electromagnetic methods for underground pipeline detection. It applies signals to pipelines through a transmitter, generating a secondary magnetic field around the pipelines. By using a receiver to measure the secondary magnetic field, the system accurately determines the position, depth, and direction of the pipelines. There are various structures in oil and gas stations, especially with a large number of grounding points, which can cause significant signal interference to the RD8000. Therefore, the equipment can only be used in simple working conditions, and in complex stations it is not accurate. At present, the question of how to troubleshoot failures of a flexible anode system has become a difficult problem restricting the application of flexible anodes in actual production.

This study presents a field case and associated methodology to investigate a flexible anode failure in a complex cathodic protection environment, such as a station. To find the reason for poor protection in the station, a series of on-site troubleshooting tests were conducted, including on/off potential and corrosion potential measurements, feeding current tests, grounding resistance measurement, and an excavation test.

## 2. Test Methods

### 2.1. Survey of the Station and Automatic Potential Control Rectifier

The buried steel pipelines in the station have a diameter of 600 mm, wall thickness of 6.4 mm, and pressure of 4 MPa, and the buried parts of the pipelines are coated with a viscoelastic and heat-shrinkable sleeve. The regional cathodic protection system for buried pipes and other metal structures with them in the station was designed and constructed in 2012, adopting the impressed current protection mode, with constant potential instrumentation of 6 circuits (5 circuits are put into operation and 1 circuit is on standby), and the 5 auxiliary anodes are all made of MMO/Ti flexible anode material (hereinafter referred to as flexible anode). Five circuits were designed to protect buried pipes in different areas, including the valve group area, inlet process area, sub-transmission branch process area, filtration area, pipe cleaning area, and tank area.

**Figure 1:**
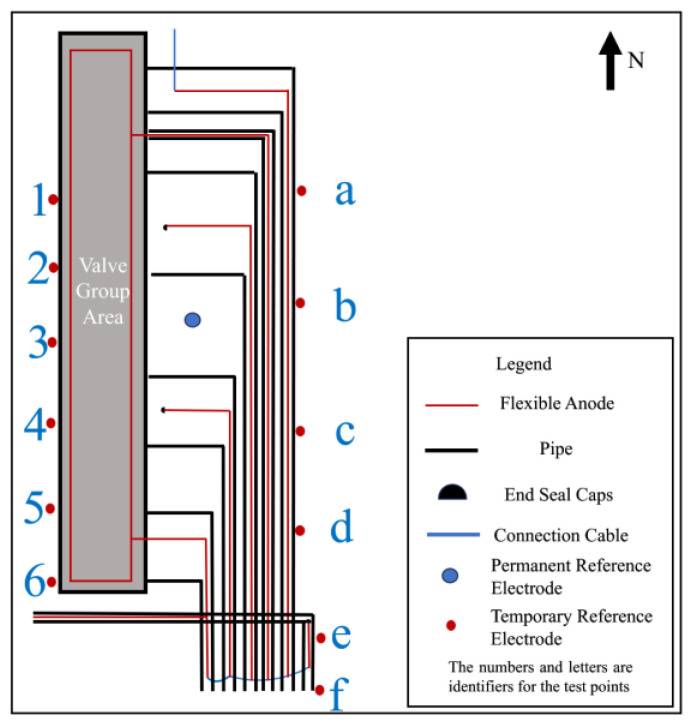
Test site distribution.

After the flexible anode cathodic protection system of the station was put into operation, a comprehensive test of the cathodic protection effectiveness was carried out. The operation parameters of the 5 circuits’ automatic potential control rectifier in the station were investigated. The test and survey results are shown in Table 1. The anodes of the same circuit are electrically connected by a cable, and there is no electrical connection between the anodes of different circuits. Circuit 1′s automatic potential control rectifier exhibited higher measurements for potential, output voltage, and output current compared to other circuits. Additionally, the circuit resistance of Circuit 1 was lower. It was found that except for the pipes in the valve group area protected by Circuit 1, the polarized potentials (off-potentials) in other areas were more negative than −0.85 V_CSE_, satisfying the criteria of ISO 15589 [17]. However, in the valve group area, most potentials were more positive than −0.85 V_CSE_. The long-term reference electrode used for the potential data of Circuit 1′s Automatic Potential Control Rectifier and the temporary reference electrode used for off-potential testing are marked in Figure 1.

### 2.2. Measurement of Cathodic Protection on and off Potential and Corrosion Potential

Figure 1 presents the relative positional relationship between the flexible anode and pipeline in the valve group area where anomalies exist. According to the distribution of pipelines and flexible anodes, a series of test sites were selected for the pipeline and anode potential measurements in the valve group area, and the distribution of test sites is shown in Figure 1. Copper/saturated copper sulfate electrode was selected as a reference electrode. Before measuring, ensure the proper functioning of the cathodic protection system and full polarization of the pipe. Place the copper sulfate reference electrode on moist soil directly above the pipeline, following the temporary reference electrode position in Figure 1. Utilizing the automatic synchronous on/off function of the Automatic Potential Control Rectifier, off-potential testing was conducted. The optimal on/off period is 12 s/3 s [18]. Connect the multimeter to the pipe and the copper sulfate electrode, adjusting the multimeter to the appropriate gear to record the on-potential and off-potential. The measured off-potential is the pipe-to-ground potential after eliminating the IR drop caused by the protection current. Then, 24 h after turning off the automatic potential control rectifier, keep it in the off state, repeat the above test steps, and record the value as corrosion potential.

### 2.3. Soil Resistivity

Soil resistivity is a basic parameter of the cathodic protection system. The resistivity test of the soil near the anode burial depth in the underground area of this station site was conducted by using Wenner’s four-pole method [19]. Three test points were randomly selected in the station, each point was tested three times, and the final result was averaged. The average soil resistivity was found to be about 300 Ω·m at 2 m depth.

### 2.4. Feeding Test

**Figure 2:**
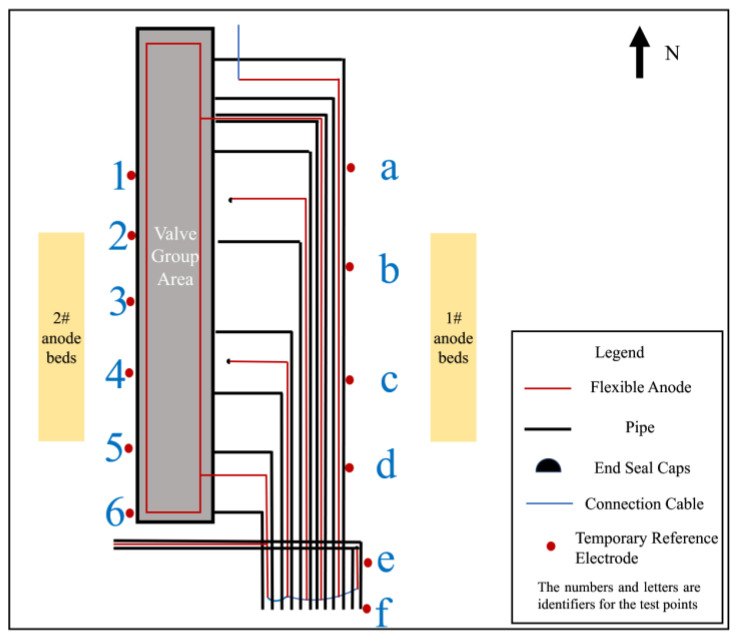
Feeding test anode ground bed burial location.

The feeding test can be used to determine the current demand for the cathodic protection of buried pipelines in the existing station [20]. The feeding test is used to establish a simulated cathodic protection system in the station, temporarily protect the buried facilities in different zones, detect the potential distribution and corresponding current demand of the buried facilities, and analyze and determine the current loss points, shielding areas, and interference that seriously affect the cathodic protection effect in the station [21]. Due to the more positive cathodic protection potential in the valve group area, an on-site feeding test was carried out to confirm whether the cathodic protection current was insufficient in this area. The change in protection effect was assessed by increasing the protection current through the temporary anode ground bed. According to the station space, pipeline distribution position and protection potential distribution, the pre-embedded position and quantity of the temporary feeder anode ground bed were determined. According to the characteristics of the station, 2 sets of temporary anode ground beds were set up, the material was Φ150 mm × 1500 mm high silicon cast iron, and the location of the anode ground beds is shown in Figure 2. After the anode ground beds were buried, the soil was returned to the ground, compacted, and wetted with water to reduce the soil resistivity near the anodes. After the temporary anode ground bed installation was completed, the valve group area pipeline was energized to apply current for polarization. After 1~2 h of polarization, testing of the on and off potential of the pipeline was carried out to evaluate the polarization offset.

### 2.5. Measurement and Calculation of Anode Grounding Resistance

The flexible anode grounding resistance of the ground bed was calculated and tested in the field. Flexible anode grounding resistance was tested using a JinChuan (Nanjing, China) ZC-8 grounding resistance meter [18].

The calculation of the grounding resistance of the flexible anode was performed according to Equation (1) [22].

(1) $$R=\frac{\rho }{2\pi l}\ln\frac{l^{2}}{tD}$$

Equation: buried horizontal anode length is *l*, m; anode diameter is *D*, m; anode burial depth is *t*, m; and *ρ* is soil resistivity is Ω·m.

It is known that the soil resistivity at the burial depth is 300 Ω·m, the anode diameter is 0.038 m, and the anode burial depth is 2 m. The flexible anode length can be calculated by bringing the anode grounding resistance *R* into Equation (1).

### 2.6. Polarization Curve Testing

**Figure 3:**
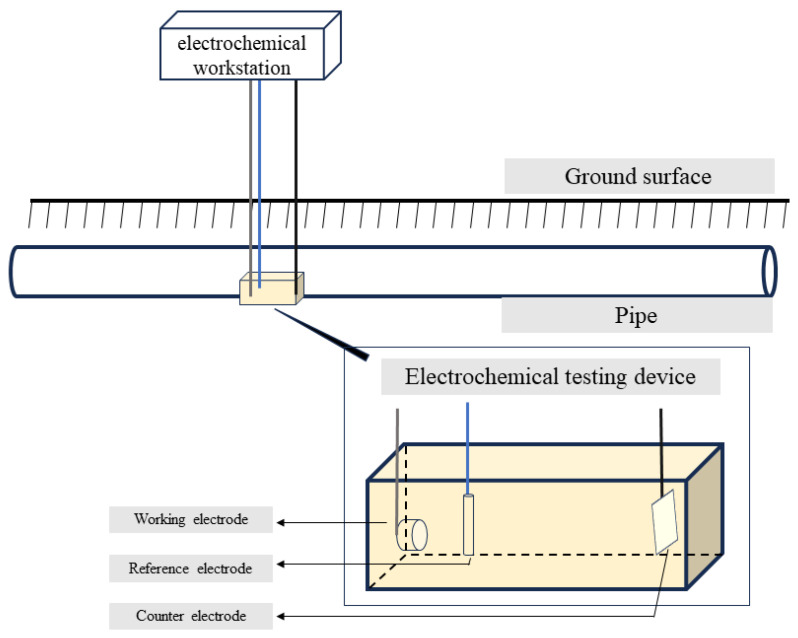
Diagram of on-site polarization curve testing apparatus.

To obtain the polarization characteristics of the pipeline, a polarization testing apparatus was installed on-site. The apparatus included a BaoSteel (Shanghai, China) Q235 steel specimen with an area of 6.5 cm^2^ as the working electrode, a mixed metal oxide plate as the counter electrode, and a saturated copper sulfate electrode as the reference electrode, as shown in Figure 3. Subsequently, a small-scale excavation was carried out to bury the testing apparatus at the same depth as the pipeline, supported by rods and backfilled with the original soil. After 1 h of settling, the polarization characteristics testing commenced. The polarization curve was tested using a Gamry (Warminster, PA, USA) Reference 3000 electrochemical workstation with a scanning rate of 1 mV/s and a scanning range of −1.5 V_CSE_~0.1 V_CSE_ [23].

## 3. Field Tests and Experimental Results

### 3.1. Measurement Results of Cathodic Protection Potential in the Valve Group Area

#### 3.1.1. Measurement Results of On and Off Potential

**Figure 4:**
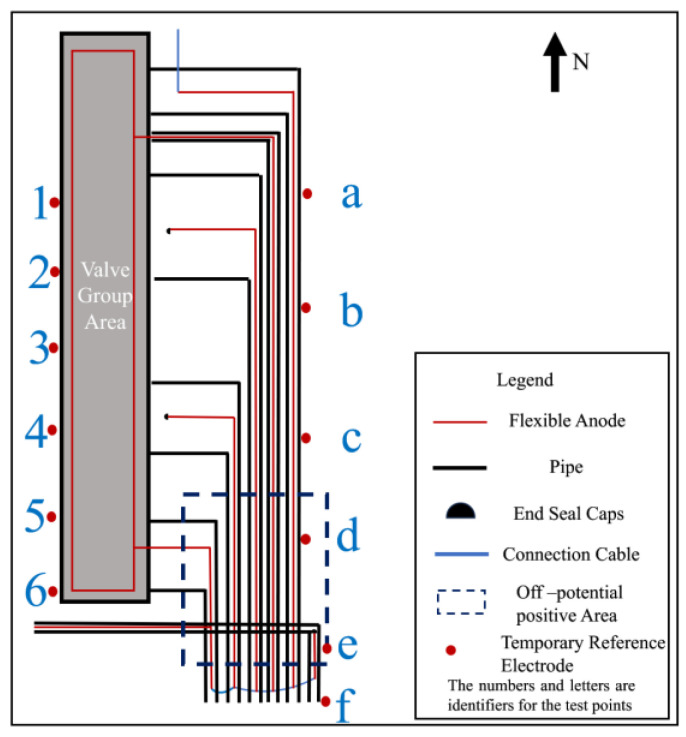
Most positive area of off-potential.

On and off potential were measured on the pipelines in the valve group area and the results are shown in Table 2. From the test results, the valve group area on-potential was in range −1453~−972 mV_CSE_, but the overall off-potential was more positive, and the pipeline was in a state of being under protection. From the numerical distribution of each test site, the most positive off-potential area is concentrated in the south side of the valve group area, as shown in Figure 4.

#### 3.1.2. Results of Corrosion Potential Measurements

Corrosion potential measurements were made after the power of the complete cathodic protection system was turned off for 24 h. The measurement results are shown in Table 3. The corrosion potential of the pipeline at test sites 2~5 and b~e in the valve group area was positively shifted to the range of −350~−170 mV_CSE_, which is more positive than that of the normal steel pipeline in the soil [17]. The most positive corrosion potential area is coincident with the most positive corrosion potential area.

### 3.2. Feeding Test Results

In order to exclude the automatic potential control rectifier failure factors, a feeding test was performed in the valve group area. Adjust the DC regulated power supply output current to be sufficiently polarized for data measurement. The measurement of on and off potential was carried out according to the test site locations shown in Figure 1. The anode ground bed used for the feeding test is shown in Figure 2 at position 1#, and the results are shown in Table 4; the feeding test is carried out again using the anode ground bed at position 2# in Figure 2, and the test results are shown in Table 5. As can be seen from the following table, the off-potential of the pipeline at all test points in the two feeding tests did not satisfy the cathodic protection criterion of −850 mV_CSE_. Even the off-potential of the pipeline at test sites c and d near the anode bed of 1# and test sites 3 and 4 near the anode bed of 2# are still more positive at −450 mV_CSE_. Consistent with the previous test pattern, the south side of the valve group area is still the area with the largest positive shift of the off-potential.

## 4. Problem Analysis and Solutions

From the analysis of the experimental results in the previous section, it is concluded that the off-potential and corrosion potential of the valve group area are more positive, especially in the south side of the valve group area where the problem is most serious. Preliminary speculation in the valve group area is that there is a positive grounding body interference, or flexible anode and pipeline electronic shorting. In order to further investigate the possible causes, numerical simulation calculations and field tests were carried out.

### 4.1. Troubleshooting the Possibility of Grounding Electrode Interference in the Valve Group Area

**Figure 5:**
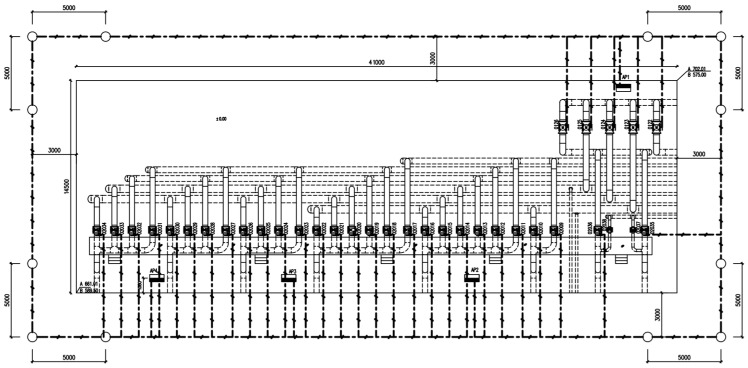
Distribution of grounding electrodes in the valve group area.

Figure 5 is the valve group area grounding electrode distribution diagram. The station valve group area grounding system is mainly divided into two kinds: the vertical grounding pole thickened galvanized steel angle and the horizontal grounding pole thickened galvanized flat steel. We have conducted tests and the ground electrode corrosion potential is at −550 mV_CSE_. These ground electrodes are connected to the pipe and do not cause the corrosion potential of the pipe to move forward to −200 mV_CSE_. The grounding electrode interference is not the root cause of the valve group area protection potential deviation [17].

### 4.2. Numerical Simulation Calculation and Analysis

**Figure 6:**
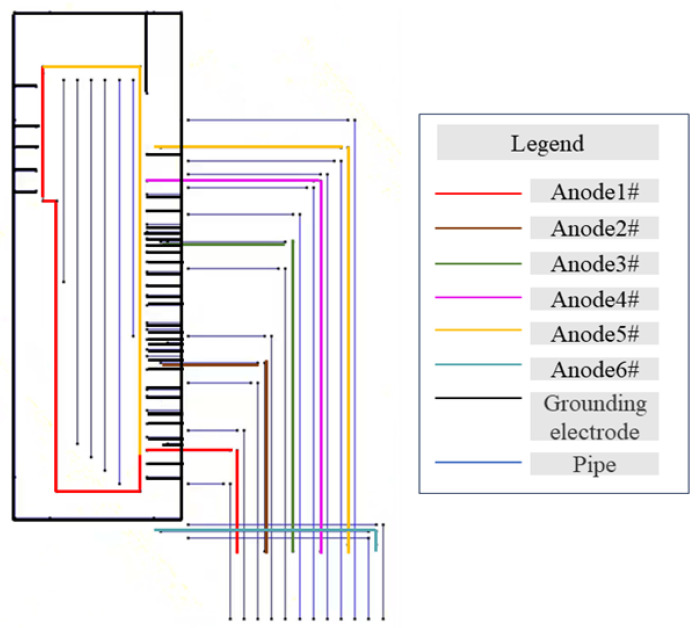
Valve group area pipeline and anode geometry modeling.

Numerical simulation calculations have been used in the optimization of the design of cathodic protection for pipelines [24]. In view of the possible electronic shorting of anodes and pipelines in the valve group area, numerical simulation was utilized for further investigation. A geometric model was established based on the distribution of buried pipelines, flexible anodes, and grounding electrodes in the valve group area mentioned above, and the model was solved using the boundary element method. The model is considered to be a steady-state cathodic protection system during the solution process, and the pipe is surrounded by a uniform soil medium, which satisfies the static field theory, which can be described by the Laplace equation, $\frac{\partial^{2}E}{\partial x^{2}}+\frac{\partial^{2}E}{\partial y^{2}}+\frac{\partial^{2}E}{\partial z^{2}}=0$, where *E* is the distribution of the cathodic protection potential [25]. The model developed is shown in Figure 6.

To solve the Laplace equation, the boundary conditions of the anode surface, cathode surface, and insulating surface have to be determined, where the anode boundary is set as a constant current boundary, $\sigma \frac{\partial E\left(x,y,z\right)}{\partial n\left(x,y,z\right)}=-q$, the anode current flows only in the soil medium, the soil surface (that is, the ground surface) is regarded as an insulating surface, and the normal current density is 0: $\sigma \frac{\partial E\left(x,y,z\right)}{\partial n\left(x,y,z\right)}=0$.

**Figure 7:**
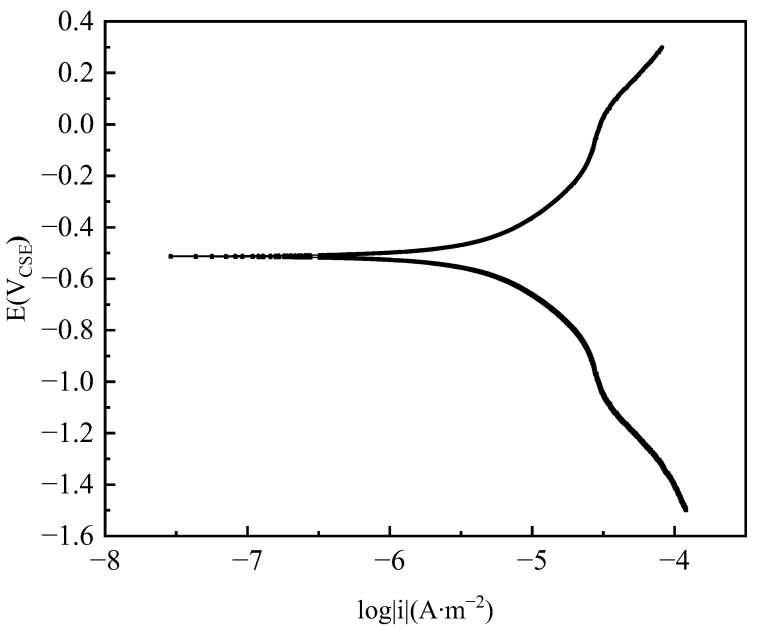
Polarization curves of pipeline steel in soil simulants.

The electrochemical reaction occurs on the surface of the pipeline, and its boundary conditions shall be determined according to the cathodic polarization characteristics; that is, the relationship between polarization potential and polarization current density, and the polarization curves of the pipeline have been measured in the actual environment, as shown in Figure 7. 

**Figure 8:**
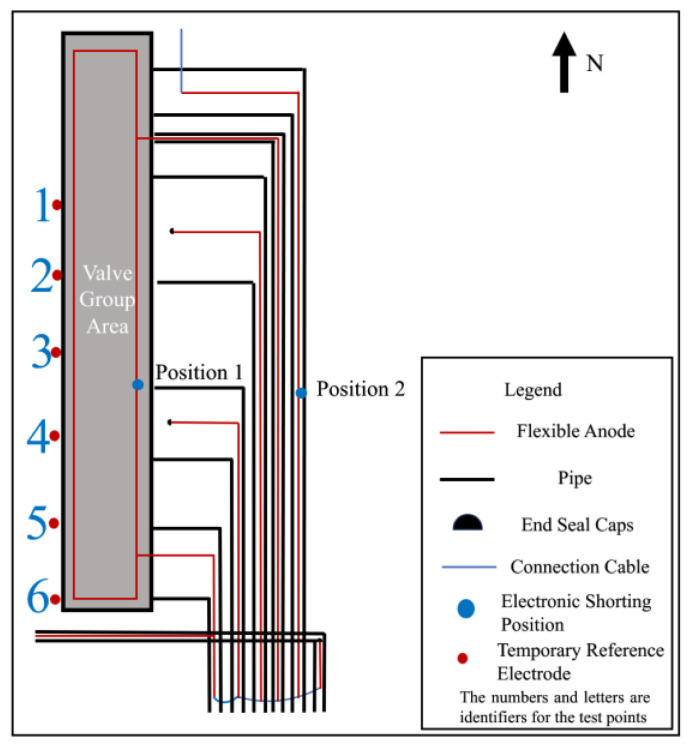
Setting of electronic shorting and measurement site locations for numerical simulation.

**Figure 9:**
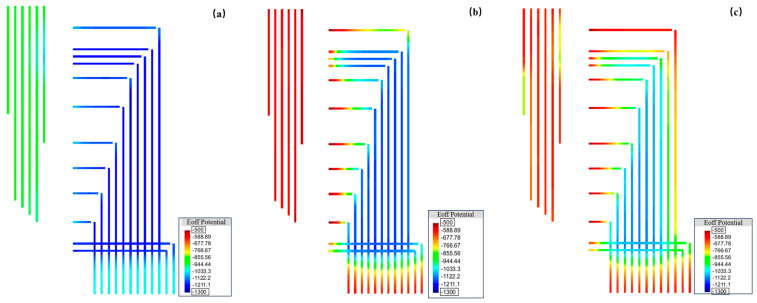
Potential distribution cloud diagrams of the flexible anode in three working conditions: (**a**) normal; (**b**) electronic shorting to pipeline at position 1; (**c**) electronic shorting to pipeline at position 2.

In order to explore the electronic shorting patterns between the anode and the pipeline, calculations were performed for three scenarios: normal operation of the anode without electronic shorting, anode and pipeline electronic shorting only at position 1, and anode and pipeline electronic shorting only at position 2. The electronic shorting positions are illustrated in Figure 8. The corresponding potential distribution cloud diagrams are presented in Figure 9, and the polarization potentials at positions 1 to 6 are shown in Table 6.

The result of Figure 9 can simulate the potential distribution across the valve group area when a location of the flexible anode is electronically shorted from the pipe. Firstly, the scenario without shorting in the entire valve group area was simulated, with the rectifier output current set to 10 A (about 1.68 A for each flexible anode). The results are shown in Figure 9a, indicating that all pipelines are in a generally good protective state. When a short circuit occurs at position 1, maintaining the rectifier output current at 10 A, the results in Figure 9b show a forward shift in potential near the left shorting location. The potentials at test points 1–6 shift from around −870 mV_CSE_ to −560 mV_CSE_, with a maximum shift of approximately 300 mV_CSE_. At this point, the potentials on the right side of the area remain below −850 mV_CSE_. Similarly, when a short circuit occurs on the right side at position 2, keeping the rectifier output current constant at 10 A, the results in Figure 9c indicate a change in the pipeline potential distribution compared to shorting position 1. In this case, the potentials on the left side shift to approximately −620 mV_CSE_ to −810 mV_CSE_, while the potentials near position 2 on the right side shift to above −750 mV_CSE_. Due to assumptions in the simulation, such as constant soil resistivity, and limitations in the test conditions, the calculated results may differ from the actual field test outcomes. However, based on the overall pattern of potential changes, it can be inferred that the shorting of flexible anodes to pipelines may cause a certain degree of positive potential shift in pipelines near the shorting point. This shift is likely due to the positive anode body being electrically connected to the pipeline, forming a local electrochemical pair that absorbs a significant amount of protective current. Based on the results in Figure 9b,c, it is hypothesized that when shorting occurs near position 1 on the left side of the area, the pattern of potential changes is more similar to the results of the previous feeding test. Therefore, it is assumed that the shorting between flexible anodes and pipelines occurs near position 1. 

### 4.3. Troubleshooting the Possibility of Anode Electronic Shorting in the Valve Group Area

#### 4.3.1. Excavation Tests for Electronic Shorting Exclusion

**Figure 10:**
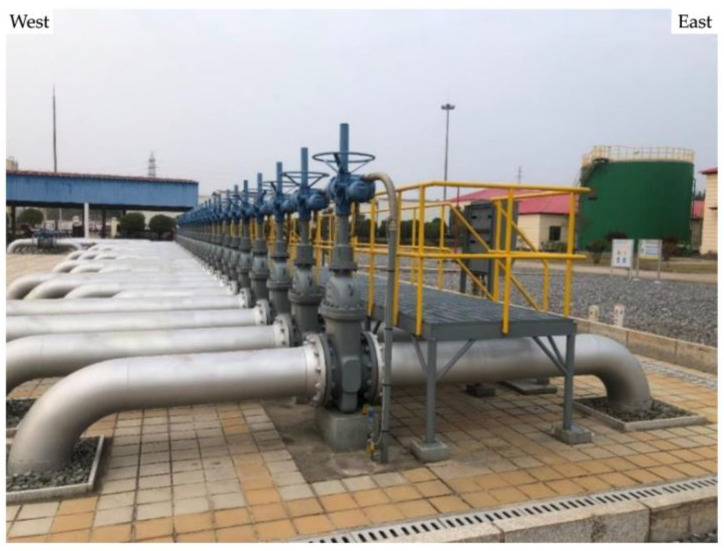
Site map of the valve group area.

**Figure 11:**
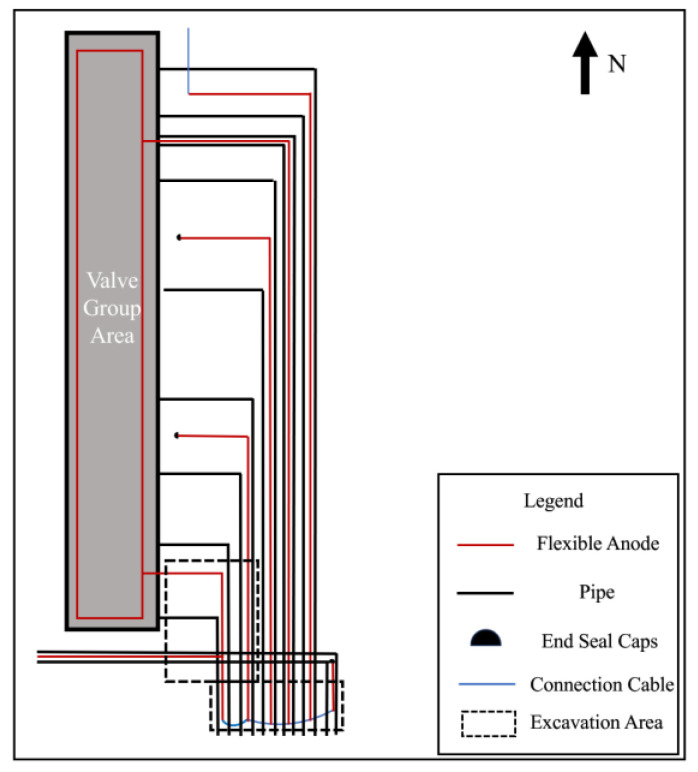
Schematic diagram of the valve group area excavation.

Figure 10 shows the site photographs of the valve group area. In order to investigate whether there is an electronic shorting connection between the anode and the pipeline, local excavation operations were carried out in the area shown in Figure 11 on the south side of the valve group area, where the pipeline off-potential and corrosion potential are most positive, to carry out a local electronic shorting exclusion test.

#### 4.3.2. Measurement of Anode On-Potential, Off-Potential, and Grounding Resistance

**Figure 12:**
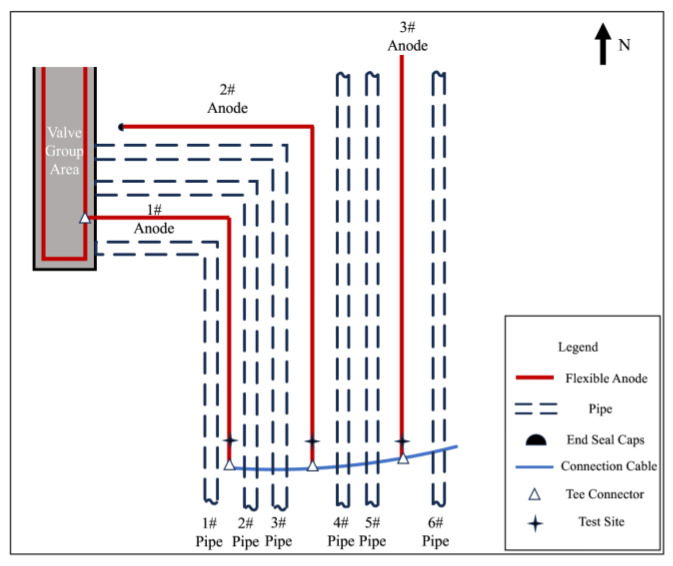
Schematic of the pipeline and anode after excavation.

After excavation, the anode-on-potential and anode-off-potential of flexible anodes were measured, and the test points are shown in Figure 12, and the test results are shown in Table 7. In normal conditions, when the anode is energized, anodic polarization occurs, causing positive shifts in both the on-potential and off-potential of the anode. The off-potential of the anode should be more positive than at its corrosion potential, but lower than the anode-on-potential. However, the anode-off-potential of the 1# anode and 2# anode was too negatively shifted, and it is presumed that there exists an electronic shorting connection with the pipeline at the 1# or 2# anode. The 3# anode has a more negative anode-on-potential and a more positive anode-off-potential, which is not in accordance with the principle of anodic polarization. The results of the grounding resistance measurement are shown in Table 8. The 3# anode grounding resistance of 6.4 Ω, and other anodes compared to the resistance value, is large; presumably, in the 3# anode there is a broken cable problem.

#### 4.3.3. The 3# Anode Cable Breaking Location Investigation

**Figure 13:**
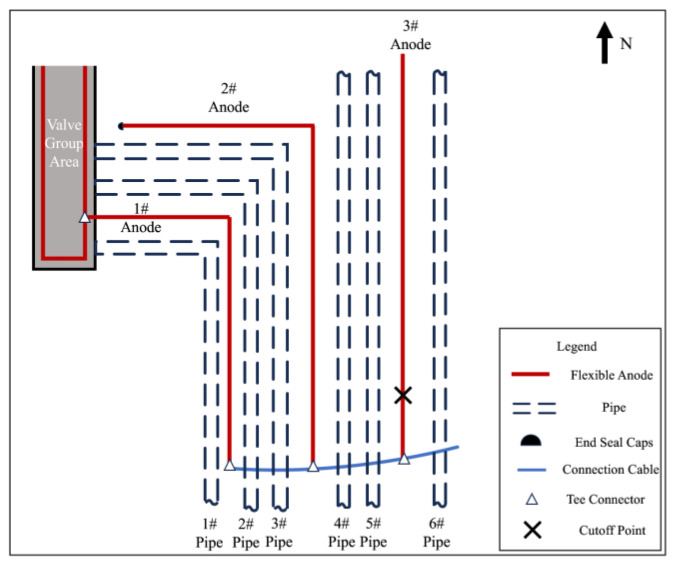
Schematic diagram of the 3# anode cutting position.

**Figure 14:**
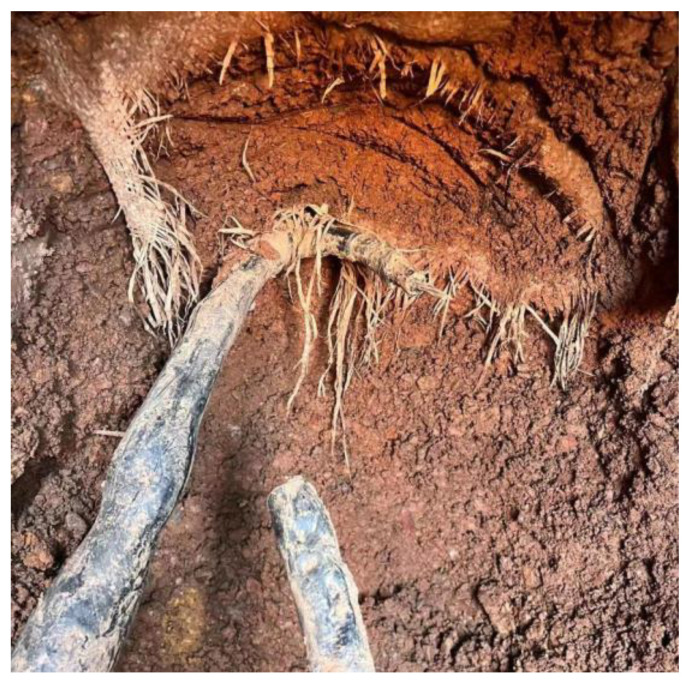
The 3# anode disconnection location.

The 3# anode was cut off at the position shown in Figure 13, and Table 9 shows the results of anode grounding resistance measurements on the two cut-off points, respectively, after cutting off the 3# anode. The value of anode grounding resistance on the north side of the cut-off point is larger, and it can be seen from Equation (1) that the shorter the length of anode *l*, the larger the grounding resistance *R*, which leads to speculation that there is a disconnection on the north side of the cut-off point of the 3# anode. Using Equation (1), it can be inferred that the location of the break point is 9.7 m. Finally, the location of the break point was found by excavation, as shown in Figure 14. After measuring, the location of the 3# anode cable breakage from the cut-off point was 10 m. After reconnecting the 3# anode cable breakage, the problem of cathodic protection potential not reaching the standard in the valve group area was not effectively improved.

#### 4.3.4. Anode and Pipeline Electronic Shorting Location Exclusion

**Figure 15:**
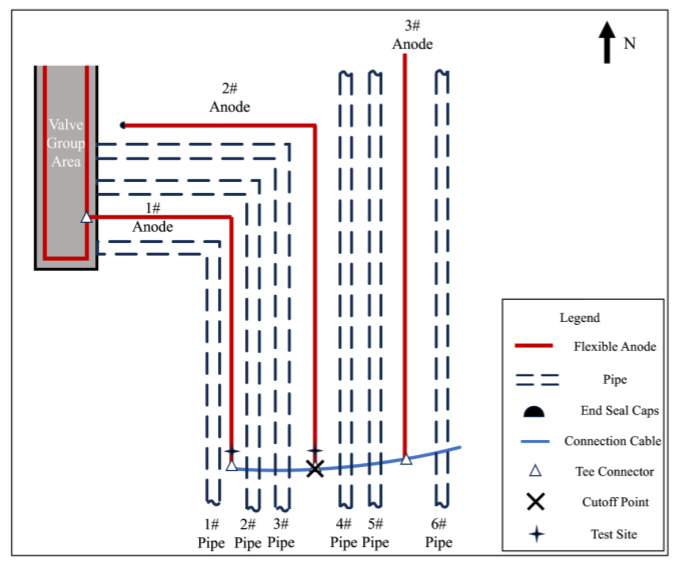
Schematic diagram of the cut off at the T-joint of the 2# anode.

The T-joint position of the 2# anode was cut off, and the cut-off position is shown in Figure 15. Table 10 shows the measurement results of corrosion potential before and after cutting off the 1# anode and the 2# anode. It can be observed that the off-potential of the 2# anode shifted positively from −180 mV_CSE_ to −100 mV_CSE_, while the off-potential of the 1# anode shifted negatively from −180 mV_CSE_ to −450 mV_CSE_. After disconnecting from the 2# anode, its corrosion potential approaches the corrosion potential of a normal anode, but for the 1# anode, it shifted even more negatively. Therefore, our focus for the investigation was narrowed down to anode 1#. 

**Figure 16:**
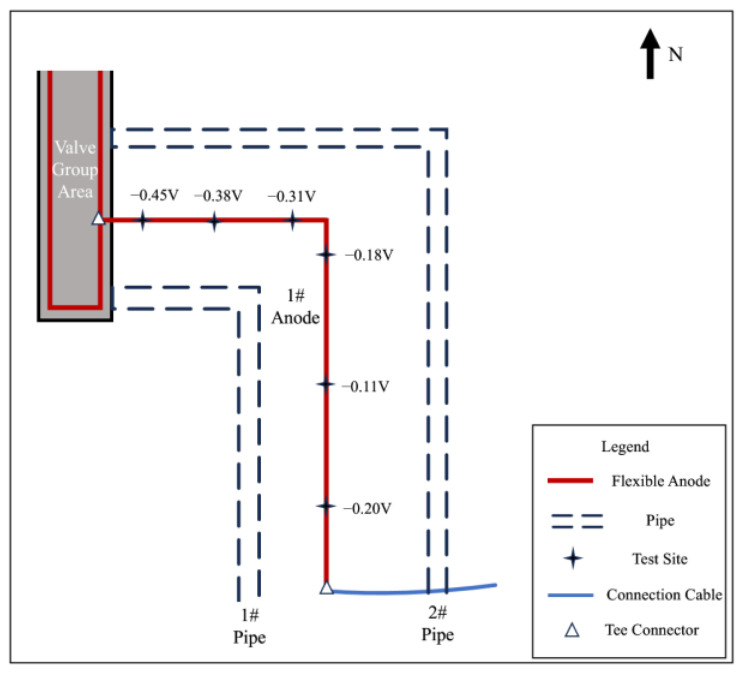
The 1# anode corrosion potential and location of the measurement point.

Select measurement points along the direction of the 1# anode cable laying for corrosion potential measurement and then mark the measurement results on the corresponding position in Figure 16. The closer to the anode in the valve group area shown in the figure, the larger the negative shift of the corrosion potential is, and it is presumed that there is an electronic shorting connection between the 1# anode and the underground buried pipeline in the valve group area.

**Figure 17:**
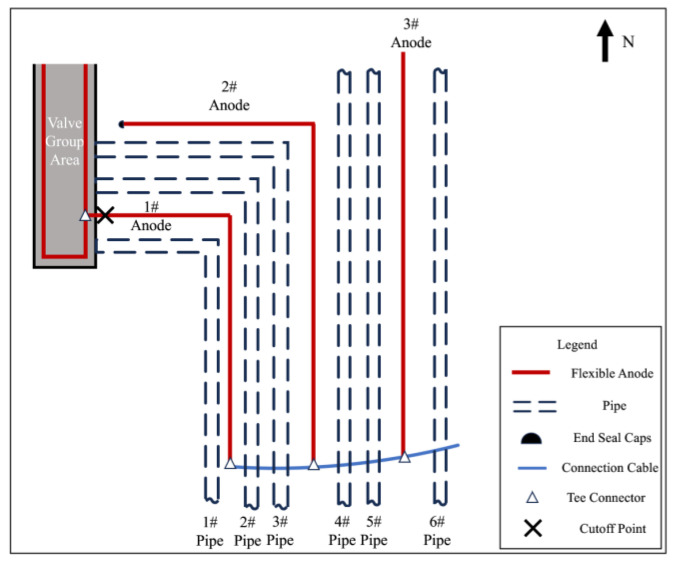
Schematic diagram of 1# anode cut off.

To further determine the electronic shorting position of the anode and the pipeline, the 1# anode was cut off close to the valve group area, and the cut-off position is shown in Figure 17. Carry out insulation sealing treatment for the end of the 1# anode at the cut-off point. Place the rest of the anode cut-off and break point in accordance with the cathodic protection system wiring diagram to restore the connection and carry out the insulation anti-corrosion treatment. After 24 h of cut-off, the potential of the pipeline on the west side of the valve group area was measured according to the test site labeled in Figure 1. Table 11 shows the comparison results of the corrosion potential of the pipeline before and after the treatment of the electronic shorting point, and Table 12 shows the comparison results of the on and off potential of the pipeline before and after the treatment of the electronic shorting point. After treatment, the corrosion potential of the pipeline is restored to normal, and the off-potential of the pipeline is reduced to below −850 mV_CSE_. From this, it can be judged that the 1# flexible anode and the underground pipeline in the valve group area electronic shorting, resulting in the previously measured potential, are the mixed potential of the pipeline and the flexible anode.

Through the on-site test, on-site troubleshooting methods are established for the two failure forms of flexible anode and pipeline electronic shorting and broken cable, respectively, which are summarized as follows:

1. Test method for detecting the electronic shorting location between the flexible anode and pipeline.

Firstly, close the applied current cathodic protection system, carry out the corrosion potential distribution test, select the area where the corrosion potential of the pipe ground is more positive, and carry out the local excavation test; after digging to the flexible anode body, carry out the local disconnection test, and measure the change in the ground potential of different anode bodies before and after disconnection. The area where the potential of the anode body is positively shifted is farther away from the electronic shorting position, and the area where the potential of the anode body is negatively shifted is closer to the electronic shorting position. According to the degree of negative shift of the anode potential, the electronic shorting point was investigated and eliminated, and the system returned to normal.

2. Test method for the on-site inspection of broken flexible anode cables.

First of all, take into consideration the output voltage and output current of different circuits of flexible anode cathodic protection power supply equipment, the preliminary calculation of circuit resistance, and the preliminary judgment of the existence of flexible anode disconnection circuit based on the larger circuit resistance, according to the circuit resistance rough assessment of the effective length of the flexible anode; in the effective length, carry out the local excavation test, cut off the flexible anode with large grounding resistance, and measure the grounding resistance of the cut-off point on both sides. The grounding resistance of the anode on both sides of the cut-off point is measured. Substitute the resistance of the side with a larger resistance value into the formula for the grounding resistance of the flexible anode to deduce the length of anode, and then determine the location of the anode disconnection cable.

## 5. Conclusions

In order to investigate the failure causes of the flexible anode protection system in an actual station, field measurements on native potentials, CP potentials, grounding resistance, feeding tests, numerical simulations, and on-site excavation were carried out. The following conclusions could be obtained:

1. The main reasons for the failure of the flexible anode protection system in the actual station were the electronic shorting between the local area of the flexible anode and the buried pipeline and the broken partial flexible anode.
2. Based on potential measurements on the flexible anode and pipes, and on-site excavation, the test method for detecting the electronic shorting location between the flexible anode and pipe was proposed.
3. Based on the anode grounding resistance measurements, on-site excavation, and calculation, a test method for the on-site inspection of broken flexible anode cables was proposed.

## Figures and Tables

**Table 1:** Pipe, anode, automatic potential control rectifier parameters, and circuit resistance.

Parameters	Circuit 1	Circuit 2	Circuit 3	Circuit 4	Circuit 5
Anode length/m	480	551	394	425	605
Number of power points	5	6	4	5	7
Pipe length/m	850	964	708	805	953
Pipe diameter/m	0.6	0.6	0.6	0.6	0.6
Pipe surface/m^2^	1601	1816	1334	1517	1795
Targeted on potential/mV_CSE_ *	−2552	−1476	−1491	−1453	−1482
Measured on potential/mV_CSE_ *	−2551	−1474	−1488	−1453	−1484
Measured off potential/mV_CSE_ *	−516~−212	−1200~−850	−1200~−850	−1200~−850	−1200~−850
Output current/A	16.52	2.21	1.75	1.18	0.82
Output voltage/V	7.01	5.17	2.63	2.61	3.13
Circuit resistance/Ω	0.42	2.34	1.50	2.21	3.82

* All the potentials shown in this paper are relative to copper/saturated copper sulfate reference electrodes (abbreviated as CSE).

**Table 2:** Test site pipeline on-potential and off-potential.

West Test Site	On-Potential/mV_CSE_	Off-Potential/mV_CSE_	East Test Site	On-Potential/mV_CSE_	Off-Potential/mV_CSE_
1	−974	−516	a	−1441	−516
2	−987	−420	b	−1420	−383
3	−972	−325	c	−1329	−258
4	−1051	−274	d	−1453	−226
5	−1030	−338	e	−1419	−212
6	−985	−457	f	−1175	−639

**Table 3:** Test site pipeline corrosion potential.

West Test Site	Corrosion Potential/mV_CSE_	East Test Site	Corrosion Potential/mV_CSE_
1	−403	a	−427
2	−334	b	−324
3	−273	c	−238
4	−235	d	−173
5	−302	e	−171
6	−411	f	−568

**Table 4:** The 1# anode ground bed feeding test results.

	Test Site	On-Potential/mV_CSE_	Off-Potential/mV_CSE_
Feed input electricity currents 1.68 A	a	−1598	−633
	b	−1978	−578
	c	−2421	−403
	d	−1947	−352
	e	−1570	−293
	f	−1198	−661

**Table 5:** The 2# anode ground bed feeding test results.

	Test Site	On-Potential/mV_CSE_	Off-Potential/mV_CSE_
Feed input electricity currents 2 A	1	−1228	−468
	2	−1127	−452
	3	−1109	−340
	4	−1167	−286
	5	−1086	−338
	6	−1065	−459

**Table 6:** The off potential at positions 1~6 for electronic shorting and normal connections.

Test Site	Off Potential/mV_CSE_		
	Normal	1 Position Electronic Shorting	2 Position Electronic Shorting
1	−865	−542	−614
2	−868	−559	−636
3	−871	−561	−709
4	−874	−576	−801
5	−877	−580	−813
6	−878	−592	−813

**Table 7:** Flexible anode on and off potential.

Test Site	Anode-Corrosion Potential/mV_CSE_	Anode-On-Potential/mV_CSE_	Anode-Off-Potential/mV_CSE_
1# anode	826	274	−6
2# anode	836	512	−63
3# anode	830	442	564

**Table 8:** Anode grounding resistance.

Test Site	Grounding Resistance/Ω
1# anode	0.9
2# anode	1.6
3# anode	6.4

**Table 9:** Grounding resistance on the north and south sides after cutting the 3# anode cable.

Test Site	Grounding Resistance/Ω
North of 3# anode cut-off point	35
South of 3# anode cut-off point	9

**Table 10:** Anode corrosion potential after the 1# and 2# anodes are cut off.

Test Site	Anode Corrosion Potential/mV_CSE_	
	Before Cutting	After Cutting
1# anode	−180	−450
2# anode	−180	−100

**Table 11:** Corrosion potential of the pipeline before and after the electronic shorting joint treatment.

Test Site	Pipeline Corrosion Potential/mV_CSE_	
	Electronic Shorting Joint before Treatment	Electronic Shorting Joint after Treatment
1	−403	−487
2	−334	−534
3	−273	−574
4	−235	−514
5	−302	−503
6	−411	−546

**Table 12:** Pipeline on and off potential before and after electronic shorting joint treatment.

Test Site	Pipeline Potential before Electronic Shorting Joint Treatment/mV_CSE_		Pipeline Potential after Electronic Shorting Joint Treatment/mV_CSE_	
	On-Potential	Off-Potential	On-Potential	Off-Potential
1	−974	−516	−1050	−940
2	−987	−420	−950	−879
3	−972	−325	−1298	−1015
4	−1051	−274	−1324	−1107
5	−1030	−338	−1305	−1004
6	−985	−457	−1092	−913

## Data Availability

Data are contained within the article.
